# Pulmonary Mucormycosis: Beyond Classic COVID-19-Associated Fungal Infections

**DOI:** 10.7759/cureus.52849

**Published:** 2024-01-24

**Authors:** Rita Pereira, Sara Neves, Maria Ruão, Celina Gonçalves, Carla Teixeira

**Affiliations:** 1 Anesthesiology, Intensive Care and Emergency Department, Centro Hospitalar Universitário de Santo António, Porto, PRT; 2 Infectious Diseases Department, Centro Hospitalar Universitário de Santo António, Porto, PRT

**Keywords:** covid-19, covid-19-associated mucormycosis, rhizopus, mucor, covid 19, pulmonary mucormycosis

## Abstract

Coronavirus disease 2019 (COVID-19) is often linked to a broad range of opportunistic bacterial and fungal infections. The second wave of the COVID-19 pandemic has witnessed an unprecedented surge in mucormycosis cases, predominantly in India, while the disease remained relatively rare in Europe. The authors describe the case of a 62-year-old female patient admitted to the hospital for consolidation therapy with chemotherapy as a part of the treatment protocol for acute myeloid leukemia. During hospitalization, she was diagnosed with nosocomial COVID-19, which later progressed to respiratory deterioration. COVID-19 with bacterial superinfection was presumed, leading to the initiation of empirical antibiotic therapy. A bronchoscopy was performed several days later due to a lack of improvement, revealing an infection by the *Rhizopus microsporus *complex. Despite antifungal treatment, the patient experienced an unfavorable clinical course and ultimately died. Given the high index of suspicion required to diagnose pulmonary mucormycosis, which can lead to delays in appropriate treatment and increase the burden of disease, the authors are aiming to enhance its awareness.

## Introduction

Mucormycosis is a rare angio-invasive fungal infection caused by a group of molds called mucormycetes [1,2]. The estimated global incidence varies from 0.005 to 1.7 cases per million people [2,3]. India is the country with the highest number of reported cases globally, with an incidence rate 80 times higher than developed countries due to environmental factors, such as exposure to spores, and chronic diseases, such as poorly controlled diabetes mellitus [2,4]. On the other hand, hematological diseases and organ transplantation are the most frequently described risk factors in developed countries. Other conditions contributing for disease development include neutropenia, immunosuppression with corticosteroids or chemotherapy, solid organ neoplasms, and iron overload. However, the disease can occur in immunocompetent individuals or persons without risk factors [3]. The clinical presentation varies according to the organ involvement, although it is well recognized that disease progression is marked by an aggressive and life-threatening course, emphasizing the critical importance of an early diagnosis and intervention [2].

COVID-19 is also associated with an increased risk of secondary fungal infections, such as aspergillosis, invasive candidiasis, and mucormycosis [1]. The pathophysiological and metabolic changes caused by SARS-CoV-2 and its therapeutic strategies have been implicated as facilitators of disease development. With the second wave of COVID-19, there was an unprecedented increase in the number of mucormycosis reports, prompting India to declare mandatory disease reporting in May 2021. Since then, although on a much smaller scale, additional cases of COVID-19-associated mucormycosis (CAM) have been reported worldwide, including in the United States and Europe [1,5,6]. To the authors' knowledge, this is the first report of pulmonary mucormycosis associated with COVID-19 in Portugal.

## Case presentation

**Figure 1:**
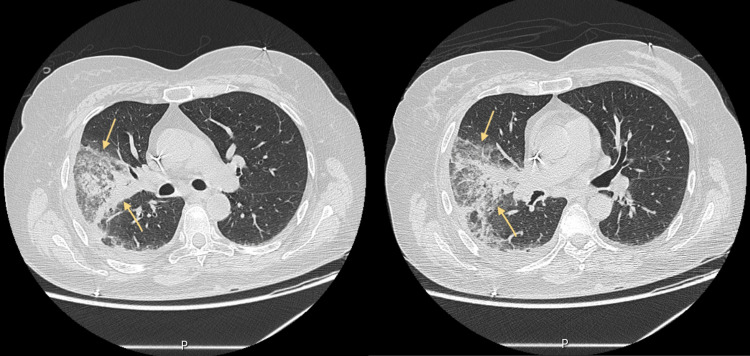
Chest computed tomography performed during ICU stay.

We present the case of a 62-year-old woman from Iran, living in Portugal for seven months with a history of acute myeloid leukemia (inversion of chromosome 16). Due to early disease recurrence, the patient underwent induction chemotherapy with complete remission (by morphologic and flow cytometric assessment) and was electively admitted to the hospital afterwards for a cycle of consolidation chemotherapy. Severe pancytopenia due to consolidation chemotherapy leads to red blood cell and platelet transfusions and filgastrim therapy. On the seventh day of hospitalization, she was diagnosed with mild nosocomial COVID-19, for which she received treatment with remdesivir. A week later, the patient developed septic shock from bacteremia originating from a urinary tract infection caused by extended-spectrum beta-lactamase (ESBL)-producing *Escherichia coli* and was admitted to the intensive care unit. Empirical antibiotherapy was started with piperacillin/tazobactam, vancomycin, and fluconazole, later adjusted to imipenem according to isolation and drug sensitivity testing. Regardless of a favorable initial evolution, there was fever recurrence on the 18th day of hospitalization associated with cough, blood-stained sputum, and respiratory failure. Chest computed tomography (CT) showed a densification in a ground-glass pattern with some areas of consolidation in the right lung (Figure 1).

In this context, the diagnosis of severe COVID-19 with secondary bacterial infection was made, a five-day dexamethasone regimen was initiated, and antibiotic therapy was escalated to ceftazidime/avibactam, vancomycin, and amikacin. Due to the lack of clinical improvement on the 23rd day, bronchoscopy was performed, which revealed multiple endobronchial lesions of a circular, irregular morphology with a vesicular appearance, suggestive of fungal infection. Those results lead to treatment with liposomal amphotericin B at a 5 mg/kg dose, increased to 10 mg/kg after isolation of a *Rhizopus microsporus *complex in bronchoalveolar lavage (BAL) culture. Despite systemic antifungal treatment, clinical evolution was unfavorable.

## Discussion

CAM is defined by the diagnosis of mucormycosis during or after the confirmation of SARS-CoV-2 infection. There is no established temporal limit as to when the mucormycosis diagnosis must be made for the disease to be considered associated with COVID-19, but it can extend up to 90 days after COVID-19 diagnosis [7].

Clinical presentation varies according to the affected region. Tissue invasion may occur at the nasal cavity, paranasal sinuses, orbits, central nervous system, and respiratory, renal, and gastrointestinal systems, and it can be disseminated [6,8]. Rhino-orbito-cerebral mucormycosis represents the main form of CAM globally and in India, while pulmonary and disseminated involvement is more frequently described in developed countries [5].

Prompt diagnosis implies a high level of suspicion, reliant on the combination of suggestive clinical presentation, compatible imagiologic findings, and histopathological and/or microbiological evidence. Imaging examinations should be appropriate to the site of concern and include brain CT scan or magnetic resonance imaging (MRI), chest/abdomen CT scan, and endoscopic procedures. Definitive diagnosis is confirmed when there is a culture isolation of the *Rhizopus microsporus* complex, positive polymerase chain reaction (PCR) in tissues or BAL, or presence of *Mucorales* hyphae in biopsy [7,9,10].

A multimodal approach is used in mucormycosis treatment that includes the use of systemic antifungal agents, surgical treatment with debridement or resection of affected tissues, and control of involved risk factors. Liposomal amphotericin B is the first-line treatment, while isavuconazole and posaconazole are reserved for refractory cases or cases of amphotericin toxicity. There is no evidence supporting combined therapy [9]. CAM treatment guidelines follow the same guidance standards [7].

In the present clinical case, in addition to acute myeloid leukemia and neutropenia, SARS-CoV-2 infection and corticosteroid treatment contributed as additional risk factors for mucormycosis. Moreover, hematological diseases dictate a preferential involvement of the lung [3]. Clinically, pulmonary mucormycosis presents an overlap of symptoms that can be attributed to the worsening of SARS-CoV-2 pneumonia, bacterial secondary infection, or other fungal infections associated with COVID-19, such as aspergillosis. The presence of hemoptysis and brown or black sputum are the most suggestive symptoms that prompt the diagnosis, in association with fever, cough, and pleuritic chest pain. Imagiologic findings are also unspecific and may include pulmonary infiltrates, parenchymal consolidation, multiple nodular lesions, pleural effusion, thick-walled cavities, mediastinal or hilar lymphadenopathy, and pneumothorax. The reverse halo sign is often associated with mucormycosis but was only described in up to 18% of cases [11,12]. Combined medical and surgical treatment is more effective than pharmacological treatment alone; however, in this case, only systemic antifungal therapy could be offered because the patient’s clinical condition did not allow lung resection. The mortality rate of CAM is estimated between 9% and 76%, strongly influenced by the site of infection and geographical distribution. Pulmonary mucormycosis is the form of the disease with the highest mortality rate, justified by the high index of suspicion required to diagnose it and unspecific clinical manifestations and imaging, leading to inevitable delays in appropriate treatment and worst outcomes [6].

## Conclusions

To the authors' best knowledge, this is the first case of pulmonary mucormycosis reported in Portugal, which is in line with the evidence that this is not a common disease in the European context. This case also aims to highlight the challenges and pitfalls of diagnosing the pulmonary form, since it does not present clinical or radiological findings that differentiate it from severe COVID-19 infection, bacterial superinfection, or other forms of fungal involvement.

Therefore, this report hopes to raise awareness among physicians about the presence of this entity, which could lead to early diagnosis and treatment, potentially reducing morbidity and mortality rates in affected patients, especially in countries with a low incidence of the disease.
